# S100B Levels in Patients with Type 2 Diabetes Mellitus and Co-Occurring Depressive Symptoms

**DOI:** 10.1155/2018/5304759

**Published:** 2018-11-18

**Authors:** Panagiota Katsanou, Nikolaos Tentolouris, Despoina Perrea, Spyridon Katsanos, Vasiliki Ntova, Virginia Antrian, Panagiotis Konstantopoulos, Antonios Politis

**Affiliations:** ^1^Diabetes Center, First Department of Propaedeutic and Internal Medicine, Laiko General Hospital, National and Kapodistrian University, Medical School, Athens, Greece; ^2^Laboratory of Experimental Surgery and Surgical Research N.S. Christeas, National and Kapodistrian University, Medical School, Athens, Greece; ^3^Heart Failure Unit, Department of Cardiology, Attikon University Hospital, National and Kapodistrian University of Athens, 1 Rimini St, 12462, Athens, Greece; ^4^Department of Endocrinology, Nikaia General Hospital, Greece; ^5^Mental Health Center, G. Gennimatas General Hospital, Athens, Greece; ^6^First Department of Psychiatry, Eginition Hospital, National and Kapodistrian University of Athens, Vassilissis Sofias Ave 72-74, 11528, Athens, Greece

## Abstract

Depression is a comorbid condition in patients with Type 2 Diabetes mellitus (T2DM). S100B, a glia derived protein, is linked to depression and has been suggested as a biomarker for depression outcomes in several populations. However, to date there is no data about S100B levels and depression in patients with T2DM.* Objective*. We hypothesized that S100B serum levels are increased in patients with T2DM and recently diagnosed, drug-free depressive symptoms, and could be used for the diagnosis of depression in T2DM.* Methods*. Overall 52 patients (62 ± 12 years, female 66, 7%) with no history of depression deriving from the Diabetes out-patient clinic of our University Hospital underwent a one-to-one interview with a psychiatrist and filled a self-assessment (Zung) questionnaire. Serum S00B levels were compared between 30 (63±12 years, female 66, 7%) diabetic patients without depressive symptoms vs 22 patients (62 ±12 years, female 68, 2%) with T2DM and depressive symptoms.* Results*. There was no difference in serum levels of S100B between patients with T2DM without depressive symptoms vs diabetic patients suffering from depressive symptoms (2.1 (1.9-10.9) pg/ml vs 2.4 (1.9-14.8) pg/ml, p=0. 637+). Moreover, linear regression analysis did not show any association between lnS100B levels and depressive symptoms (*β* = 0.084, 95% CI 0.470-0.871, and p=0.552), Zung self-assessment score (*β* = 0.048, 95% CI -0.024-0.033, and p=0.738), and other patients' characteristics.* Conclusions*. In patients with T2DM there is no correlation between S100B serum levels and newly detected mild depressive symptoms. The brain biochemistry pathways of depression in T2DM warrant further investigation in a larger scale population.

## 1. Introduction

Depression is a common comorbid condition in patients with Type 2 Diabetes mellitus (T2DM) [1] with important clinical significance. Studies have shown that patients with coexistent T2DM and depression appear to be at higher risk for poor glycemic control [2], myocardial infarction [3], and early mortality [4].

Several surveys have been conducted in an effort to describe the underlying mechanism of depression in T2DM, including the inflammatory theory [5], the hypothalamic-pituitary-adrenal (HPA) axis dysregulation theory [6], and the reduced neuroplasticity theory [7].

S100 calcium-binding protein b (S100B) is a molecule deriving from glia with a specified role in glial disorder. It appears to have important activity in cellular shape and intracellular communication [8]. In the general population the loss of neuroplasticity due to glial cells alterations supports the neurodegenerative hypothesis of depression [9]. Apart from the connection to neuroplasticity S100B is connected to inflammation as well [10]. The connection can be attributed to the interaction with Receptor for Advanced End (RAGE) products [11] and the upregulation of proinflammatory enzymes [12]. In some studies S100B are reported higher in depression when studied in general population [13]. Furthermore, it is recently discovered that the effect of antidepressant medicine could be the upregulation of neurogenesis especially in the adult hippocampus, which can be expressed by S100B values [14], supporting the hypothesis that S100B seems to be a marker of antidepressive treatment outcomes. Although T2DM is associated apart from inflammation, with impaired neurogenesis and decreased synaptic plasticity [15], the clinical significance of S100B is yet to be investigated in depressive patients with T2DM.

In this study we examined for differences in serum S100B concentrations between subjects with and without depressive symptoms in a cohort of patients with T2DM and for potential associations between S100B serum levels and metabolic as well as depression parameters.

## 2. Subjects and Methods

All patients derived from the Diabetes outpatient clinic of “Laiko” University Hospital. They were all adults >18 years old, diagnosed with T2DM according to the American Diabetes Association (ADA) criteria [16], and either on pharmaceutical therapy or on diet. Details from the patients' history, including micro- and macrovascular diabetes-related complications were obtained by interview and from their medical files and previous discharge letters.

Strict exclusion criteria were practiced as follows:

- Type 1 Diabetes Mellitus
- Diabetes complications which cause severe pain or a low quality of life (diabetic neuropathy, a history of diabetic foot ulcer and/or amputation, frequent hypoglycemic episodes, and diabetic retinopathy causing vision loss)
- Chronic kidney disease stage ≥3 (GFR < 59/ml/min/1.73 m)
- History of known malignancies and/or hematological diseases and/or autoimmune diseases and/or current treatment with corticosteroids
- A psychiatric history or patients under any psychiatric medication
- History of head trauma of any kind
- Pregnancy

## 3. Psychiatric Evaluation

All patients consented to fill a self-assessment questionnaire, the Zung Self-Rating Depression Scale (ZSDS). The ZSDS is designed to assess clinical depression with 20-item positively or negatively worded questions on a four-level Likert-type scale. It addresses somatic, affective, and cognitive aspects of depression. It is translated and validated in Greek with good validity [17]. Scores <50 are considered nondepressed; 50-59 mildly, 60–69 moderately, and >70 severely depressed [18].

On the same day the participants were evaluated by a psychiatrist, who was blind to the results of the self-assessment questionnaire score. Major and minor depressive disorders according to Diagnostic and Statistical Manual of Mental Disorders, Fourth Edition (DSM-IV) criteria, were assessed using the Mini-International Neuropsychiatric Interview (MINI), which is a short diagnostic structured interview [19]. Major depressive disorder was diagnosed when at least one core symptom (loss of interest or depressed mood) was present with at least four other symptoms: change in appetite or significant weight change, sleep disturbances (such as insomnia or hypersomnia), psychomotor disorders (such as agitation or retardation), loss of energy or fatigue, feelings of guilt or worthlessness, declined ability to concentrate or think or presence of indecisiveness, and finally suicidal thoughts or plans. Participants with one core symptom and one to three other symptoms were diagnosed with a minor depressive disorder. The term “depression” in this study refers to subjects with either major or minor depressive disorder, as described in the current literature when patients with Diabetes Mellitus were evaluated for depression [20]. A questionnaire [21] (Hamilton scale 17) was additionally filled by the psychiatrist and severity of depressive symptoms was estimated according to the scale [22].

## 4. Clinical Evaluation

A full clinical evaluation was performed following the psychiatric interview. Body weight was measured early in the morning with subjects in the fasting state and light clothing without shoes using a flat scale (Tanita WB-110MA, Japan), and height was measured in a stadiometer (Seca Mode 220, Germany). BMI (Body Mass Index) was calculated as weight (Kg) divided by height (m)^2^. Waist circumference was measured at the midpoint between the lower margin of the last palpable rib and the top of the iliac crest, using a stretch-resistant tape. Hip circumference was measured around the widest portion of the buttocks, with the tape parallel to the floor. Systolic and diastolic blood pressure was measured twice, 2 min apart, in a sitting position, and the average of the two was recorded, using an OMRON HEM-907XL device (Kyoto, Japan), as previously described [23].

## 5. Blood Sampling

A full blood examination was deducted the same day. Freshly drawn blood samples were used for determination of glucose, lipids, liver enzymes, and creatinine levels using an automatic analyzer, after an 8-hour fasting. Low-density lipoprotein (LDL) cholesterol was calculated using the Friedewald formula. HbA1c was measured using high-performance liquid chromatography. Serum samples were collected, separated by centrifugation at 3000 rpm for 10 min at 4C and refrigerated at -80C for further bioanalysis. In S100B and Tumor Necrosis Factor-a (TNF-a), a protein linked to depression through inflammation [24], serum levels were measured. The investigators performing the measurements were blind to the results of the other examinations.

High-sensitivity human enzyme-linked immunosorbent assay (ELISA) kits were used to measure Tumor Necrosis Factor (TNF)—a (MILLIPLEX MAP Human Metabolic Hormone Magnetic Bead Panel—Metabolism Multiplex Assay, Cat. No. HMHEMAG-34K-04, Millipore). The concentrations of human S100B in serum were measured with an enzyme-linked immunosorbent assay kit, according to protocols provided by manufacturers (Human S100B Elisa Kit, Cat. No. EZHS100B-33K, Millipore). The results were expressed in pg/mL.

All patients signed written and informed consent. The study was approved by the local hospital ethics committee and was carried out according to the Declaration of Helsinki.

This research did not receive any specific grant from funding agencies in the public, commercial, or not-for-profit sectors.

## 6. Statistical Analysis

Data were analyzed with the package SPSS (SPSS 17.0, Inc., Chicago, USA). Continuous variables were categorized normally or nonnormally distributed according to Kolmogorov-Smirnov test and visual inspection of the histograms. Data were presented as mean± standard deviation (normally distributed) or median ± interquartile range (nonnormally distributed) or as percentages in case of categorical data.

Patients were dichotomized in those with depressive symptoms and without depressive symptoms (0 or 1 respectively) according to the psychiatric evaluation. Continuous variables between 2 groups were then compared with unpaired Student *t* test when normally distributed or the Mann-Whitney test otherwise. Categorical variables were compared with *χ*^2^ or Fisher-exact test as appropriate.

Linear regression analysis was performed to evaluate the relation of lnS100B with clinical characteristics and laboratory findings of patients. The coefficient of correlation (r) and the 95% confidence interval (CI) was calculated. A p< 0.005 was considered statistically significant

## 7. Results

The mean age of patients was 62 ± 12 years. Females were 66.7% of the studied population. Depressive symptoms were diagnosed in 42.3% of the overall studied population. Demographic and laboratory characteristics of the population are presented in Table 1. Patients without depressive symptoms were more likely to have a smaller Zung score as compared to those with depressive symptoms (37 ± 9 vs 53 ± 4, respectively, p<0.001). There was no patient with severe symptoms (Hamilton score ≥ 19), one patient was with moderate depression (Hamilton score 14-18), and patients with mild depressive symptoms (Hamilton score 8-13) were 21. There were, however, no other statistical significant differences among characteristics of nondepressive and depressive patients (Table 2). There was no correlation of lnS100B values and depressive symptoms (r=0.084, CI:-0,470-0.871, p=0.552). Also, lnS100B values did not correlate significantly with any demographics and clinical characteristics of patients (Table 3).

## 8. Discussion

The results of this study indicate that S100B serum levels did not differ between nondepressive and depressive patients with T2DM. To our knowledge it is the first study examining for differences in S100B serum levels between nondepressive and depressive patients with T2DM.

S100B is already correlated to depression through two mechanisms, as described in the current literature. The first one is through inflammation. S100B is a well-known ligand to Receptor for Advanced End products (RAGE) and this interaction leads to the production of proinflammatory cytokines, such as Interleukin 6 protein (IL6) and Tumor Necrosis Factor (TNF) [25]. Depression is already connected to inflammation [26] possibly through the impact of chronic stress which is a possible link in diabetes-related depression [27]. The second mechanism is through dysregulated neuroplasticity. The term neuroplasticity actually refers to the capacity of neural system to adapt to the internal and external environment and future changes [28]. Disturbed neuroplasticity is described in depressive patients either as an anatomical sign, located on hippocampus, or as a biochemical disorder, through decreased concentration of neurotrophic factors [29]. Disturbed neuroplasticity is a possible underlying condition promoting depression in diabetic population [30]. S100B is considered to be a neuroplasticity related biomarker [31].

The strict exclusion criteria practiced in our study reinforce our findings. Firstly, S100B levels can be altered by antidepressive treatment in animal-based and in human studies. Antidepressants seem to influence the secretion of S100B in the mouse [32] and rat [33] hippocampus and in humans via the serotonergic system [34]. All participants in the present study were newly diagnosed and drug-free patients with depressive symptoms. Secondly, depressive patients with End Stage Renal Disease (ESRD) have increased S100B serum levels compared to nondepressives [35]. In the current study the presence of stage 3 Renal Kidney Disease was an exclusion criterion in order to omit any direct toxic effect of uremia on the brain cells. Moreover, patients with diabetic neuropathy were also excluded from the study, since there is an ongoing hypothesis that gliosis accompanying diabetic neuropathy may be associated with relevant markers of neurodegeneration [36]. Furthermore, S100B can be expressed in case of traumatic or nontraumatic brain injury. Mathematical models suggest that serum S100B levels above 350 ng/L(pg/ml) indicate brain damage [37]. In our study, the serum S100B levels were lower than 350 pg/ml. This result confirms that brain damage is excluded as a potential source of S100B production, as pointed in the exclusion criteria above. Finally, serum S100B levels could also be influenced by extra cranial S100B expression, since it can derive from several peripheral tissues such as white fat, skeletal muscle, or heart [8]. In the current study the two groups were matched for BMI and comorbidities in order to avoid confounders.

The assessment of depressive symptoms in the present study was based on a clinical interview (M.I.N.I.) interview by a psychiatrist, which is the gold-standard method, using additionally the Hamilton rating scale [38], which is an established tool to identify depression and symptoms' severity [39]. Furthermore, we used ZSDS, which is a self-assessment test that can quantitate the depressive symptoms and also eliminate the observer-scorer bias [40]. There are some concerns that ZDSD may not be as accurate and valid for detecting people with depression, especially since it is possible to under- or overestimate symptoms in patients with mild and moderate depression [41]; however in this study ZSDS appears to be adequate as a tool for depression diagnosis and severity, as described through the literature [42].

S100B has already been associated with depression in the general population in small scale studies with inconsistent results. The majority of the literature reveals that depressive patients appear to have higher S100B levels compared to healthy controls [13, 43–45]. This elevation could be an indication of axon growth during synaptogenesis, as a result of depressive symptoms.

We have to address some differences in the design of these studies compared to ours. Rothermoundt et al. investigated patients with a more severe form of depression and a higher Hamilton score (mean HAM-D>25) compared to ours and an unknown history of renal function contrary to our study, where all patients with stage 3 Renal Disease were excluded [43]. Moreover, more than 50% of recruited inpatients in the study of Schroeter et al. presented with recurrent episodes of depression [13]. In the present study on the other hand, patients from the outpatient clinic had no history of depression. Polyakova et al. investigated 27 subjects with minor depressive episode patients compared to healthy controls [44]. The mean age of the recruited population was different compared to our study (mean age 70 years vs mean age 62) and a potential non brain source of S100B was not ruled out. Grabe HJ et al. demonstrated that the presence of a depressive episode was significantly associated with an elevation of the cerebrospinal fluid concentration of S100B in a study comparing 11 depressive inpatients to 11 healthy inpatients controls [45]; however there was no information concerning the patients' comorbidities. On the contrary another small scale study revealed no correlation between S100B levels and depression in the general population. Interestingly, a history of depression was not an exclusion criteria and patients under current depressive episode of duration between 5,41 and 9,87 months participated in the investigation [46].

This is the first study to our knowledge that compares S100B levels between patients with T2DM with and without depressive symptoms. S100B is expressed from astrocytes and this expression is considered to be a protective event in response to a destructive process. Indeed, S100B seems to be a signal of neuron outgrowth and survival promotion [47] and is associated with restored neuroplasticity [48]. In the current study, s100B levels of T2DM patients with depressive symptoms compared to patients with T2DM without depressive symptoms did not differ, that being an indication that S100B is not expressed as expected from the glia. This can be attributed to a potential dysregulation of neuroplasticity in patients with T2DM compared to other populations, such as patients with End Stage Renal Disease, where the elevation of S100B serum levels could be the result of the accumulation of uremic toxins [35].

Taking under consideration that in the general population patients with higher S100B levels might profit more from certain antidepressants that act preferentially on the serotonergic system and influence the inhibition of serotonin reuptake, while those with lower S100B levels can benefit from different modes of action [49], we accentuate the necessity for further studies to evaluate S100B levels before and after antidepressive treatment in this specific population.

Surprisingly, we did not find any association between S100B levels and demographic characteristic or laboratory findings of our patients. Given the small number of studies addressing S100B and depression it is possible that there are more unknown pathophysiological pathways that influence S100B kinetics.

There are some limitations in this study. Firstly, the research was conducted on a small population and additional studies are undeniably required to clarify the clinical role of this biomarker in depressive patients with T2DM. Secondly, S100B levels were exclusively measured in blood, whereas cerebrospinal fluid analysis would provide additional and important information. Finally, our newly diagnosed depressive patients suffered mainly from a “mild” form of depression, which could be of clinical significance, necessitating the further evaluation of S100B in more serious forms of depression in T2DM.

## 9. Conclusion

S100B serum levels did not differ between patients with T2DM and newly diagnosed mild depressive symptoms compared to those without depressive symptoms in a small population. The exact clinical role of S100B in patients with T2DM needs further evaluation.

## Figures and Tables

**Table 1:** Overall population characteristics of patients with Type 2 Diabetes Mellitus (n=52).

Age (years)	62 ± 12

Female	35 (66. 7%)

BMI (kg/m2)	31.2 ± 5.6

Waist (cm)	104 ± 22

Current smoking	13 (25%)

Stroke/TIA	2 (3. 8%)

CAD	10 (19 2%)

Arterial Hypertension	35 (67. 3%)

MAP (mmHg)	96 ± 10

Duration of diabetes (months)	90 (36-240)

Creatinine (mg/dl)	0.8 ± 0.1

MDRD	74 (67-105)

LDL (mg/dl)	112 (77-123)

HDL (mg/dl)	47 ± 14

Triglycerides (mg/dl)	135 (96-228)

HbA1c (%)	7.1 ± 1.1

TNFa (pg/ml)	8.1 ± 2.4

S100-B (pg/ml)	2.2 (1.9-12.3)

**Medication**	

Insulin	10 (19. 2%)

Metformin	36 (69. 2%)

DPP4 inhibitors	12 (23. 1%)

Sulphonylureas	14 (26. 9%)

ACE/ARBs	27 (51. 9%)

b-blockers	18 (34. 6%)

Antiplatelet treatment	17 (32. 7%)

Statin treatment	28 (53. 8%)

Depressive symptoms	22 (42. 3%)

Hamilton (interview) rating scale score (17)	5 (3-9)

Zung self-assessment score	44 ± 10

**BMI:** body mass index, **TIA**: transient ischemic attack,** CAD**: cardiovascular disease, **MAP**: mean arterial pressure (MAP = [(2 x diastolic)+systolic]/3, **MDRD**: GFR (mL/min/1.73 m2) = 175 × (Scr)-1.154 × (Age)-0.203 × (0.742 if female) (mL/min/1.73 m2), **LDL**: low density lipoprotein, **HDL**: high density lipoprotein, **HbA1c**: glycated hemoglobin; **TNFa**: tumor necrosis factor a, **S100B**: S100 calcium binding peptide b; **DPP4 inhibitors**: dipeptidyl-peptidase 4 inhibitors; **ACE**: angiotensin-converting-enzyme inhibitor, and **ARBs**: angiotensin receptor blockers.

**Table 2:** Comparison of characteristics of type 2 Diabetes Mellitus patients without depressive symptoms vs type 2 Diabetes Mellitus patients with depressive symptoms.

	No Depressive symptoms (30)	Depressive symptoms (22)	p
Age	63 ± 12	62 ± 12	0.609
female	20 (66. 7%)	15 (68. 2%)	0.575
BMI (kg/m2)	31.3 ± 5.7	31.2 ± 5.5	0.919
Current smoking	7 (23. 3%)	6 (27. 3%)	0.757
CAD	4 (13. 3%)	6 (27. 3%)	0.183
Arterial Hypertension	22 (73. 3%)	13 (59. 1%)	0.312
MAP	98 ± 1.1	94 ± 0.9	0.185
Duration of diabetes (months)	108 (57-156)	78 (24-240)	0.933
Creatinine (mg/dl)	0.85 ± 0.17	0.88 ± 0.2	0.620
MDRD	79 ± 20	77 ± 17	0.595
LDL (mg/dl)	110 (85-123)	112 (75-130)	0.945
HDL (mg/dl)	45 ± 13	51 ± 16	0.133
Triglycerides (mg/dl)	145 (109-192)	121 (92-182)	0.394
HbA1c (%)	7.1 ± 1.3	7.0 ± 0.8	0.895
TNFa (pg/ml)	5.3 ± 2.0	6.6 ± 2.6	0.058
S100-B (pg/ml)	2.1 (1.9-10.9)	2.4 (1.9-14.8)	0.637
Insulin	4 (13. 3%)	6 (27. 3%)	0.290
Metformin	22 (73. 3%)	14 (63. 6%)	0.548
DPP4 inhibitors	5 (16. 7%)	7 (31. 8%)	0.171
Sulphonylureas	10 (33. 3%)	4 (18. 1%)	0.185
ACE/ARBs	16 (71. 5%)	11 (50%)	0.814
b-blockers	10 (33. 3%)	8 (36. 4%)	0.820
Antiplatelet treatment	9 (30%)	8 (36. 4%)	0.767
Statin treatment	16 (53. 3%)	12 (54. 5%)	0.931
Zung self-assessment score	37 ± 9	53 ± 4	**<0.001**
Hamilton (interview) rating scale score (17)	9 (8-10)	3 (2-5)	**<0.001**

**BMI:** body mass index, **TIA**: transient ischemic attack,** CAD**: cardiovascular disease, **MAP**: mean arterial pressure (MAP = [(2 x diastolic)+systolic]/3, **MDRD**: GFR (mL/min/1.73 m2) = 175 × (Scr)-1.154 × (Age)-0.203 × (0.742 if female) (mL/min/1.73 m2), **LDL**: low density lipoprotein, **HDL**: high density lipoprotein, **HbA1c**: glycated hemoglobin, **TNFa**: tumor necrosis factor a, **S100B**: S100 calcium binding peptide b, **DPP4 inhibitors**: dipeptidyl-peptidase 4 inhibitors, **ACE**: angiotensin-converting-enzyme inhibitor, and **ARBs**: angiotensin receptor blockers.

**Table 3:** Linear regression of patients' characteristics with LnS100B.

	beta	95% confidence interval	P
Age	-0.057	-0.320 -0.210	0.960
Female	-0.095	-0.943-0.458	0.502
BMI	-0.098	-0.063-0.057	0.923
Current smoking	-2.08	-1.314-0.188	0.139
CAD	0.044	-1.712-0.973	0.757
Arterial Hypertension	0.037	-0.617-0.800	0.796
MAP	-0.187	-0.051-0.010	0.185
Duration of diabetes	0.105	-0.002-0.004	0.459
MDRD	-0.202	-0.030-0.005	0.150
HbA1c	0.072	-0.211-0.357	0.610
TNF-a	-0.135	-0.700-0.199	0.341
Insulin	0.026	-0.767-0.920	0.856
Metformin	0.092	-0.484-0.951	0.516
DPP4 inhibitors	-0.072	-0.987-0.587	0.613
Sulphonylureas	-0.056	-0.896-0.601	0.694
ACE/ARBs	-0.027	-0.729-0.602	0.849
b-blockers	0.098	0.470-0.964	0.492
Antiplatelet treatment	0.113	-0.423-0.985	0.426
Statin treatment	0.169	-0.261-1.054	0.232
Depressive symptoms	0.084	-0.470-0.871	0.552
Hamilton score	0.019	-0.098-1.112	0.894
Zung self-assessment score	0.048	-0.024-0.033	0.738

**BMI:** body mass index, **TIA**: transient ischemic attack,** CAD**: cardiovascular disease, **MAP**: Mean Arterial Pressure (MAP = [(2 x diastolic)+systolic]/3, **MDRD**: GFR (mL/min/1.73 m2) = 175 × (Scr)-1.154 × (Age)-0.203 × (0.742 if female) (mL/min/1.73 m2), **HbA1c**: glycated hemoglobin, **TNFa**: tumor necrosis factor a, **S100B**: S100 calcium binding peptide b, **DPP4 inhibitors**: dipeptidyl-peptidase 4 inhibitors, **ACE**: angiotensin-converting-enzyme inhibitor, and **ARBs**: angiotensin receptor blockers.

## Data Availability

The data used to support the findings of this study are available from the corresponding author upon request.

## Supplementary Materials

Supplementary Materials Raw data regarding S100B measurements (OD readings with plate layout) is provided by the authors as supplementary material.
